# NIR hyperspectral imaging and multivariate image analysis to characterize spent mushroom substrate: a preliminary study

**DOI:** 10.1007/s00216-017-0192-2

**Published:** 2017-01-23

**Authors:** Maogui Wei, Paul Geladi, Shaojun Xiong

**Affiliations:** Department of Forest Biomaterials and Technology, Swedish University of Agricultural Sciences, 90183, Umeå, Sweden

**Keywords:** *Pleurotus ostreatus*, Sample presentation, PCA, PLS-DA, Wet vs. dry, Open vs. plastic covering

## Abstract

Commercial mushroom growth on substrate material produces a heterogeneous waste that can be used for bioenergy purposes. Hyperspectral imaging in the near-infrared (NHI) was used to experimentally study a number of spent mushroom substrate (SMS) packed samples under different conditions (wet vs. dry, open vs. plastic covering, and round or cuboid) and to explore the possibilities of direct characterization of the fresh substrate within a plastic bag. Principal components analysis (PCA) was used to remove the background of images, explore the important studied factors, and identify SMS and mycelia (Myc) based on the pixel clusters within the score plot. Overview PCA modeling indicated high moisture content caused the most significant effects on spectra followed by the uneven distribution of Myc and the plastic cover. There were well-separated pixel clusters for SMS and Myc under different conditions: dry, wet, or wet and plastic covering. The loading peaks of the related component and the second derivative of the mean spectra of pixel clusters of SMS and Myc indicated that there are chemical differences between SMS and Myc. Partial least squares discriminant analysis (PLS-DA) models were calculated and classification of SMS and Myc was successful, whether the materials were dry or wet. Peak shifts because of high moisture content and unexpected peaks from the plastic covering were found. Although the best results were obtained for dried cylinders, it was shown that almost equally good results could be obtained for the wet material and for the wet material covered by plastic. Furthermore, PLS-DA prediction showed that a side face hyperspectral image could represent the information for the entire SMS cylinder when Myc was removed. Thus, the combination of NHI and multivariate image analysis has great potential to develop calibration models to directly predict the contents of water, carbohydrates, lignin, and protein in wet and plastic-covered SMS cylinders.

## Introduction

The mushroom industry has expanded rapidly since 1990, leading to a growth in mushroom production [1]. The production of spent mushroom substrate (SMS) has also increased significantly. Starch, hemicellulose, and cellulose are the major carbohydrates of the initial substrate and are gradually degraded by mycelia during cultivation. While the mycelia grow inside and through the substrate, they build up new carbohydrates and protein in their tissues. During these processes, the substrate and mycelia are all changing in composition, depending on substrate raw material, mushroom species, and cultivation environment. SMS has been used as a potential feedstock for bioenergy [2, 3], fertilizer/soil conditioner [4, 5], and other uses [6]. The utilization of SMS is largely based on its chemical character and composition (e.g., moisture content and major organic components). Therefore, efficient methods for SMS chemical characterization can be helpful for strategic decision-making.

Near-infrared (NIR) analysis is one of the most effective methods for biological material characterization, owing to its rapidity, cost-effectiveness, non-destructive nature, and low requirements with respect to sample preparation [7]. Characterization of food products, for example, can be done by NIR spectroscopy, as water, fat, proteins, carbohydrates, and other organic matter containing C–H, O–H, C=O, and N–H bonds has high absorbance in the NIR wavelength regions (780–2500 nm) [7–9]. NIR spectroscopic analysis is usually conducted on bulk materials, from which a single NIR spectrum is obtained, and the goal is to minimize sampling errors by using averages [7, 9]. Therefore, homogeneous samples are needed for bulk NIR analysis. As for SMS of many mushroom species, they are often packed at a fixed density in containers such as the plastic bags of cylindrical shape used in this study. A direct online NIR characterization of such packed SMS cylinders would be ideal, but some technical barriers must first be tackled. The mushroom substrate in the current study (SMS of *Pleurotus ostreatus*) is wet and held together with substrate mycelia (or vegetative mycelia), which is covered by a “shell” of unevenly distributed aerial mycelium (Myc) inside the plastic cylinder bag. Some cylinders may still have fruiting bodies that emerge during the SMS-preservation period.

There are a number of factors to take into account: (1) compositional differences of the substrate, (2) errors caused by plastic and moisture, (3) distribution of Myc, and (4) shape (round or flat). Any of these factors would influence an NIR spectrum, and it is necessary to study the importance of all these factors.

NIR hyperspectral imaging (NHI) is another NIR technology for chemical characterization and has been shown to be a useful tool in food research and the characterization of other biological materials [9, 10]. The image has spatial coordinates in two dimensions as well as a wavelength coordinate, yielding a three-dimensional hypercube. This hypercube includes some depth information and allows the description of differences and gradients in the sample. Multivariate image analysis (MIA) can be used to detect and minimize background and shading errors. After this, identification and classification of the SMS and Myc regions can be done. This would be possible with little or no sample preparation. The current study investigated the potential of combined NHI and MIA for the characterization of SMS under wet and the plastic cover conditions, being the first step for a further development of the calibration modeling.

## Materials and methods

### Material origin

**Fig. 1:**
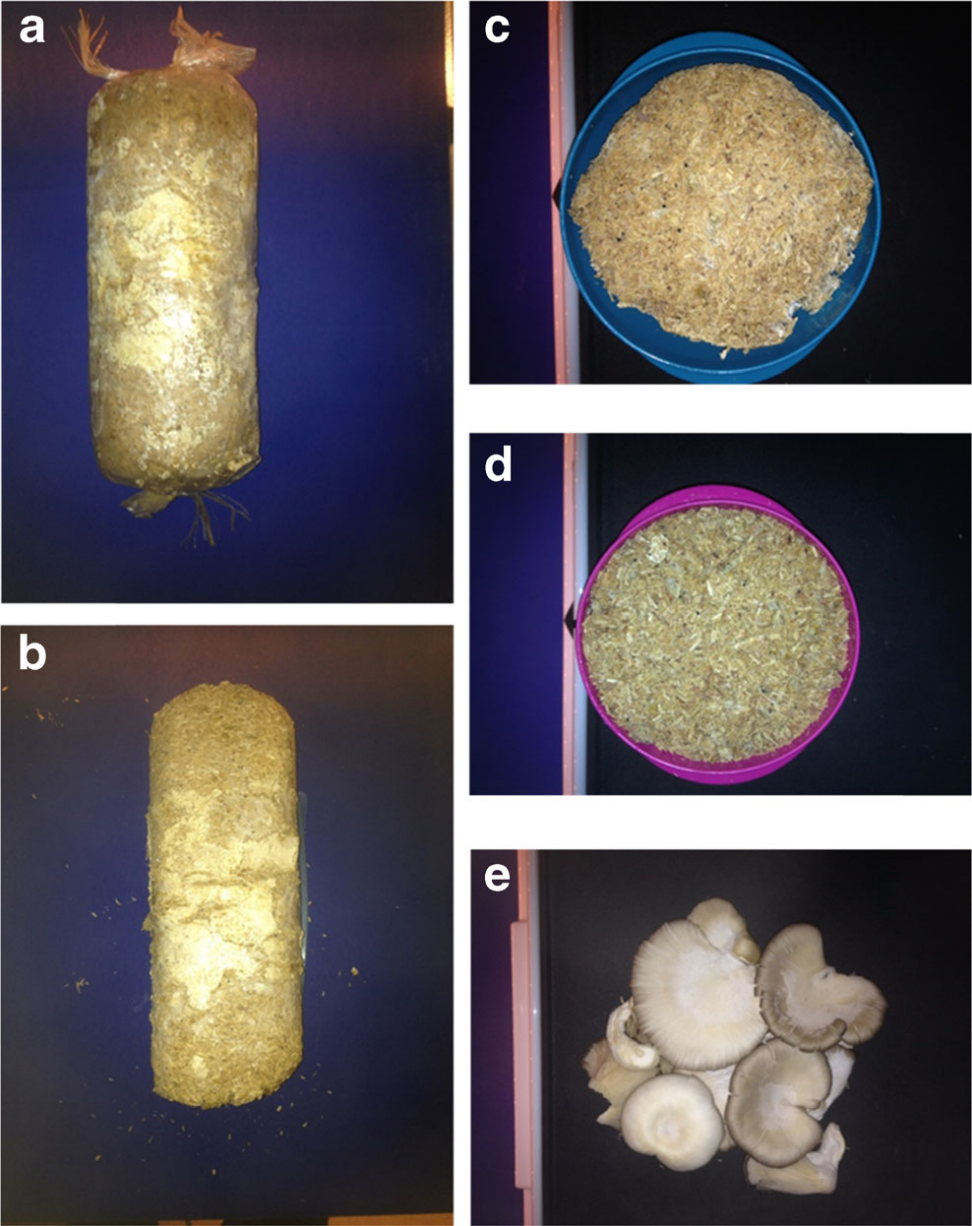
Digital images of the materials. The spent mushroom substrate (SMS) cylinder within a plastic bag (**a**) and without the plastic cover (**b**), and the aerial mycelia (the *white spots* at the surface of the cylinders); cross section of the cylinder (**c**); the bulk mixed SMS (**d**); and the mushrooms (**e**). The average diameter of the cylinders is 100 mm

The substrate material was used as cylinders (average diameter about 10 cm) prepared by a small-scale *P. ostreatus* cultivation plant in Umeå in 2015. The mushroom substrate was initially composed of 75% birch sawdust and 25% wheat grain and well mixed. After harvesting, the last flush mushroom, three SMS cylinders were collected from the same batch at the same time and preserved in plastic packing bags in a cool room before imaging (Fig. 1a). Several fruiting bodies (mushrooms) emerged from the cylinders during preservation and were collected as mushroom samples (Fig. 1e). Therefore, the studied SMS is materially composed of three fractions: substrates (remains of the degraded form of the initial substrates and inner mycelia), Myc, and mushroom. The moisture content was 64% (by drying at 105 °C for 16 h) for the fresh SMS and 90% for the fresh mushroom.

### Experimental design

The experimental design is summarized in Table 1, including four setups, 13 experiments (Exp.), and 24 runs in total. SMS and Myc were included to distinguish the influences of moisture, plastic, uneven distribution of Myc, and cylinder shape. Setup I (Exp. 1 and 2) was designed to investigate the chemical difference between SMS and Myc and the scattering effect that might be caused by the arc surface of the cylinder, while setup II (Exp. 3, 4, 5, and 6) was used to explore the influences of moisture and the plastic cover. When examining the effects of profiling on sampling representativeness that might be induced by the light penetration depth of imaging, setup III (Exp. 7, 8, 9, and 10) was used to compare the pixel spectra for SMS between the cylinder’s lateral face, cross section, and bulk mixed materials. Mushrooms (Exp. 11, 12, and 13) were applied as setup IV for use as a chemical reference to confirm the assigned spectral bonds based on widely available chemical information from previous studies [11–13].

**Table 1:** The experimental design

Setup	Exp.	Factor					Replicate
		Material	Moisture	The plastic cover	Imaging profile	Surface imaged	
I							
	1	SMS (Inc. Myc)	Dry	No	Longitudinal	Arc	2
	2	SMS (Inc. Myc)	Dry	No	Longitudinal	Flat	3
II							
	3	SMS (Inc. Myc)	Wet	Yes	Longitudinal	Arc	3
	4	SMS (Inc. Myc)	Wet	Yes	Longitudinal	Flat	3
	5	SMS (Inc. Myc)	Wet	No	Longitudinal	Flat	1
	6	SMS (Inc. Myc)	Wet	Yes	Longitudinal	Flat	1
III							
	7	SMS	Dry	No	Cross section	Flat	3
	8	SMS + Myc	Dry	No	Bulk mixture	Flat	1
	9	SMS	Wet	No	Cross section	Flat	3
	10	SMS + Myc	Wet	No	Bulk mixture	Flat	1
IV							
	11	Mushroom	Dry	No	N/A	N/A	1
	12	Mushroom	Wet	Yes	N/A	N/A	1
	13	Mushroom	Wet	No	N/A	N/A	1
Total runs							24

*SMS* spent mushroom substrate, *Myc* mycelia, *N*/*A* not applicable

### Instrumentation, sample preparation, and imaging

The instrument used for imaging was a line-scanning camera from Specim, modified by Umbio (Umbio AB, Umeå, Sweden) for measuring samples on a belt. The camera made line images of 320 pixels wide at 256 wavelengths (900 to 2500 nm). Line illumination was achieved with two rows of quartz halogen lamps. The images were corrected for camera dark current. Based on a measured Spectralon white reference, absorbance in each pixel was calculated. The OLES22 lens was provided by Specim. Synchronization of the belt speed gave square pixels. The line width of the scans was 180 mm, which resulted in pixel size of 0.6 × 0.6 mm.

The imaging process was separated into two sections, and all the images of the fresh samples were taken first. Exp. 3 was conducted by taking images from the arc surface of the first SMS cylinder, repeated for three different parts of the cylinder through rotation (Fig. 1a). The flat surface for imaging was obtained by pressing the second cylinder into a cuboid mold, which allowed three images (on different sides) to be taken for Exp. 4. After that, the plastic that covered one side of the flat surface of the cuboid was removed for Exp. 5 but replaced to cover the exposed flat surface for Exp. 6 after the removal of some detached Myc. The third cylinder was cut into three cross sections, and a flat surface on each was kept for imaging (Exp. 9, Fig. 1c); the rest of the material from the same cylinder was crumbled and mixed to yield a homogeneous sample (Fig. 1d). To conduct Exp. 10, Myc collected from the plastic bag was put on top of the mixed sample. To minimize disturbances, such as shadows and the background, efforts were made to display the fruiting bodies (mushroom) on one flat surface during imaging (Exp. 12 and 13, Fig. 1e). After drying all the materials at 45 °C with a 90% airflow rate for 96 h, the images of the dried samples (about 10% moisture content, determined at 105 °C for 16 h) were taken for Exp. 1, 2, 7, 8, and 11.

### NIR hyperspectral image analysis

Some noisy spectrum parts were removed, and 246 wavelengths from 949 to 2476 nm were used. Principal components analysis (PCA) was used to remove image background and outliers (dead or nonlinear camera pixels) [10]. PCA modeling was also used to overview the studied factors and compare their influences based on a multiple image from Exp. 1, 2, 5, and 6. After that, pixel clusters of SMS and Myc of the images were studied in a pixel score plot. By interactive brushing, score plots and score images could be related to each other. This allowed for the selection of useful components and pixel clusters and their interpretation. The averaged and cleaned spectra could be extracted by selecting and averaging the correct clusters. Mean centering and standard normal variate transformation (SNV) were chosen for the data preprocessing. Partial least squares discriminant analysis (PLS-DA) was consequently applied to confirm the cluster selection based on the results of PCA modeling [14, 15], and the models were used to verify the effect caused by the plastic cover and the representativeness of SMS spectra. Mean centering and SNV were applied here also. To explore the comparable spectra peaks that could be assigned as chemicals, the second derivative of the average spectra of SMS, Myc, and mushroom were calculated using a cubic polynomial order with 15 points in each sub-model and a distance of one point between points [16].

The Evince 2.7.0 hyperspectral image analysis software package (Prediktera AB, Umeå, Sweden) was used for image analysis (PCA and PLS-DA modeling) and mean spectrum extraction. SIMCA 13.0 (Umetrics AB, Umeå, Sweden) was used for second derivative-corrected spectrum calculation.

## Results and discussion

### PCA modeling and pixel cluster identification of SMS and Myc based on spectral information

**Fig. 2:**
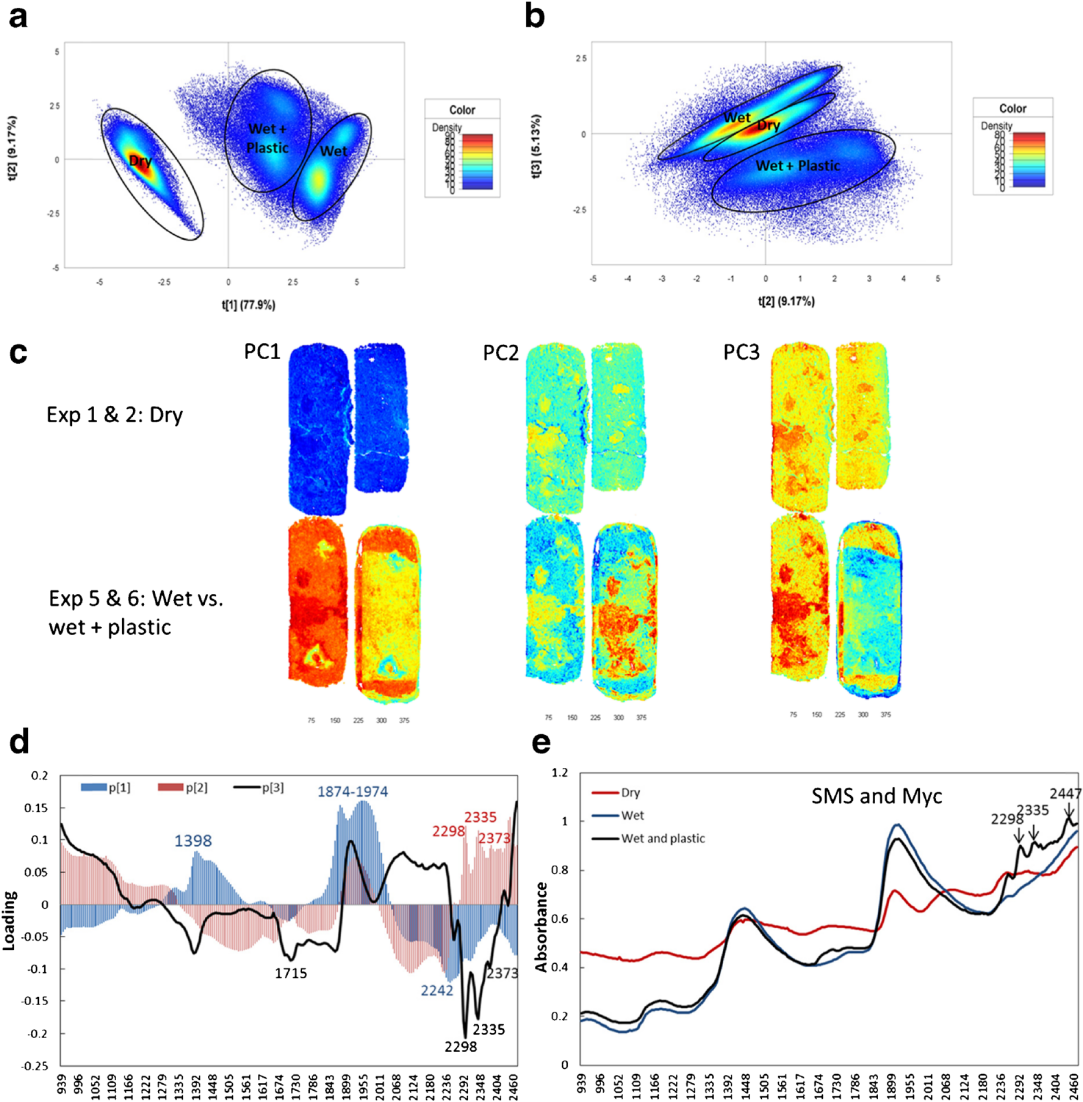
Overview principal components analysis (PCA) modeling for the comparison of studied factors. One run from Exp. 1 and 2 (dry materials), Exp. 5 (wet materials), and Exp. 6 (wet and plastic covered materials) were used to merge this multiple mosaic for the PCA modeling. **a**, **b** The pixel score plots. **c** The scores plot of the related principal components (*PC*). **d** The loading plot of the first (*blue*), the second (*red*), and the third (*dark*) components. **e** The average spectra of the pixel clusters of the dry, the wet, and the wet and plastic covered materials, including spent mushroom substrate (*SMS*) and mycelia (*Myc*)

To compare the effects of high moisture content, the plastic cover, uneven distribution of Myc, and arc or flat imaging surfaces, four images from setup I and setup II (Exp. 1, 2, 5, and 6; one run from each experiment) were merged as a multiple image for overview PCA modeling by calculating the percentage of total variance explained by each component. Five components were calculated and moisture (high absorbance at 1400 and 1900 nm) dominated the first component, which explained 77.9% of total variance (Fig. 2a, c, d). The difference between SMS and Myc dominated the second component (9.2%) and clusters of SMS and Myc were found, whether the materials were dry, wet, or wet and plastic covered. The effect caused by the plastic cover was explained by the second and the third (5.1%) components (Fig. 2b, c) which is consistent with the loading peaks (2298, 2335, 2373, and 2447 nm) of the second and the third components and the additive peaks within the average spectral of the wet and plastic covered material (Fig. 2e). The scattering caused by the round shape was explained by the fifth component, which in total explained 0.44% of the total variance (data not show). Therefore, moisture, uneven distribution of Myc, and plastic covering caused significant effects on the spectra.

**Fig. 3:**
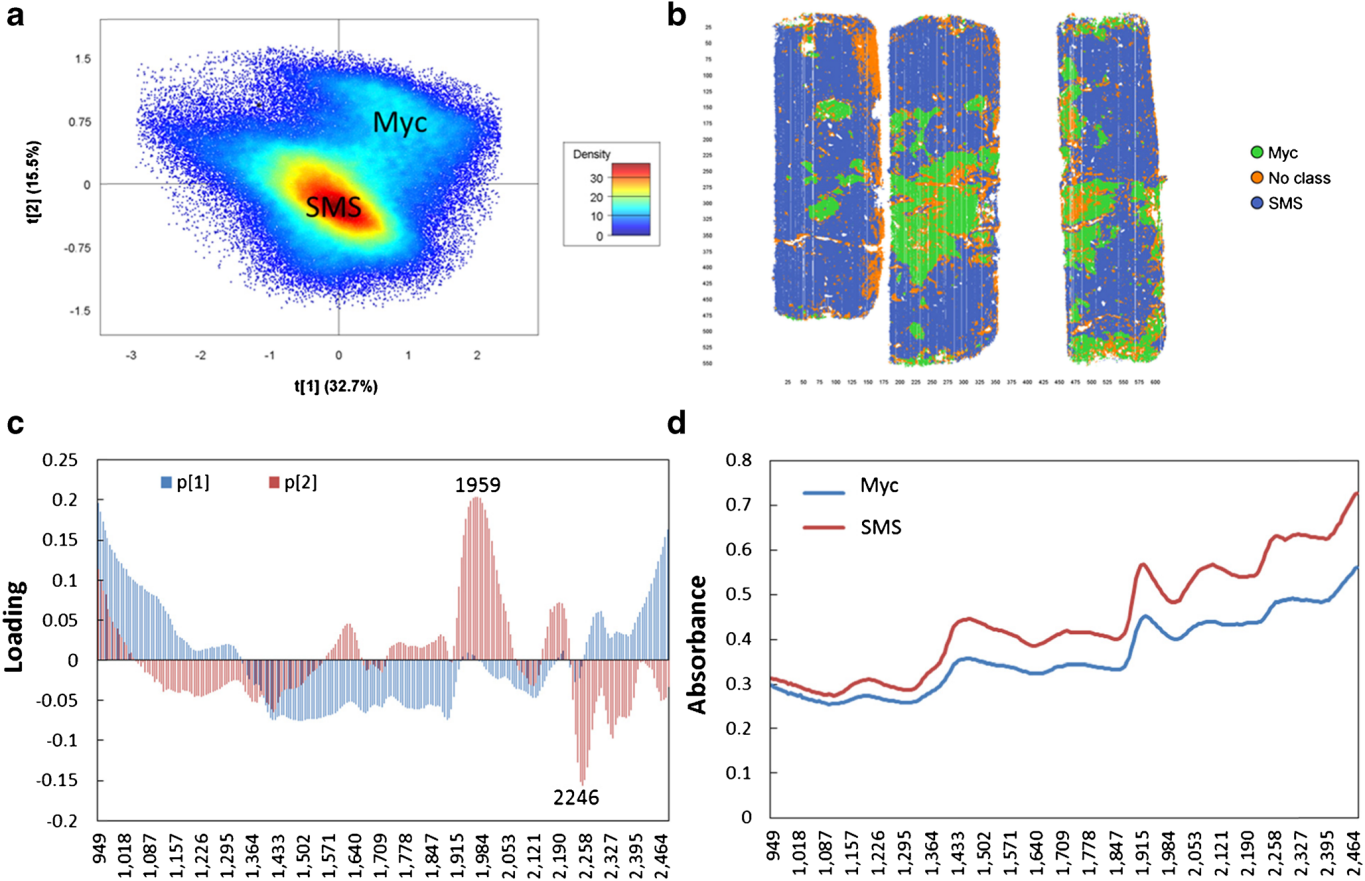
The principal components analysis (PCA) modeling for spent mushroom substrate (*SMS*) and mycelia (*Myc*) identification of the dry material. One run from Exp. 1 and two runs from Exp. 2 of setup I were applied to merge this multiple mosaic. **a** The pixel score plot. **b** The contour plot showing the result of class identification by selecting the pixel clusters within A. **c** The loading plot of the related components. **d** The mean spectra of the pixel clusters of SMS and Myc in **a**

**Fig. 4:**
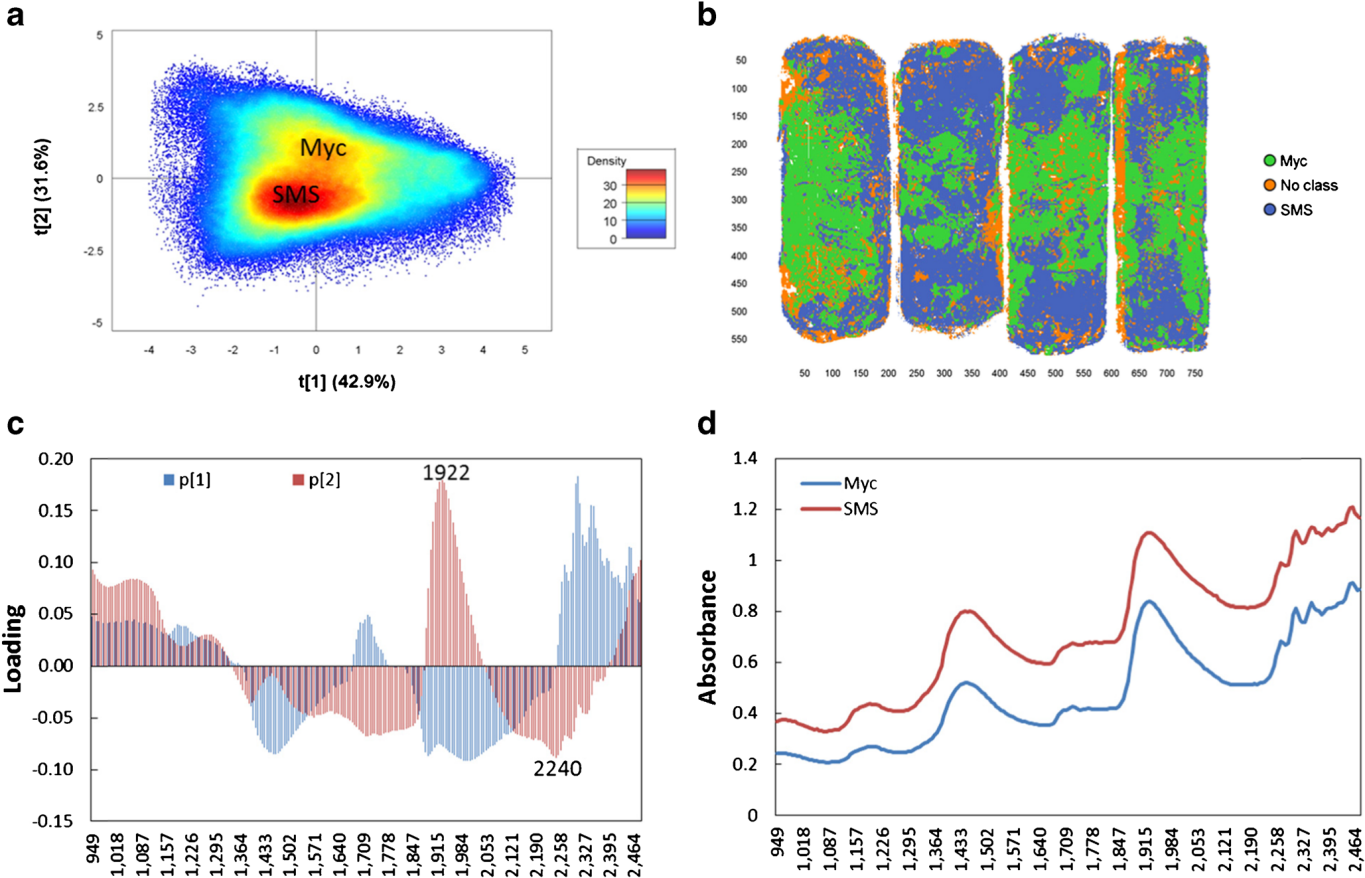
The principal components analysis (PCA) modeling for spent mushroom substrate (*SMS*) and mycelia (*Myc*) identification of the wet and plastic covered materials. Two runs of Exp. 3 and 4 were applied to merge this multiple mosaic. **a** The pixel score plot. **b** The contour plot showing the result of class identification by selecting the pixel clusters within **a. c** The loading plot of the related components. **d** The mean spectra of the pixel clusters of SMS and Myc in **a**

To explore the difference between clusters SMS and Myc, one run of Exp. 1 and two runs of Exp. 2 from setup I were merged into a multiple mosaic for PCA modeling for the dry material (Fig. 3), while two runs of Exp. 3 and 4 from setup II were merged into another multiple mosaic for the wet and plastic covered material (Fig. 4). The results show that there were clear pixel clusters for SMS and Myc and better separation for clusters was found in the dry material (Fig. 3a) than the wet and plastic-covered setups (Fig. 4a). Clear pixel clusters for SMS and Myc were also found for the wet material (Exp. 5 and 10, data not shown). According to the pixel score plots (Figs. 3a and 4a), SMS and Myc clusters were separated by the second component. High loading values of wavelengths in the loading plots (Figs. 3c and 4c) played an important role for the pixel clustering, which means the loading peaks of the second component were very important for SMS and Myc separation. The strongest positive loading peak for the dry material was approximately 1959 nm, the negative one was at 2246 nm, and the positive and negative loading peaks for the wet and plastic covered material were approximately 1922 and 2240 nm, respectively.

### Classification of SMS and Myc via PLS-DA modeling

**Fig. 5:**
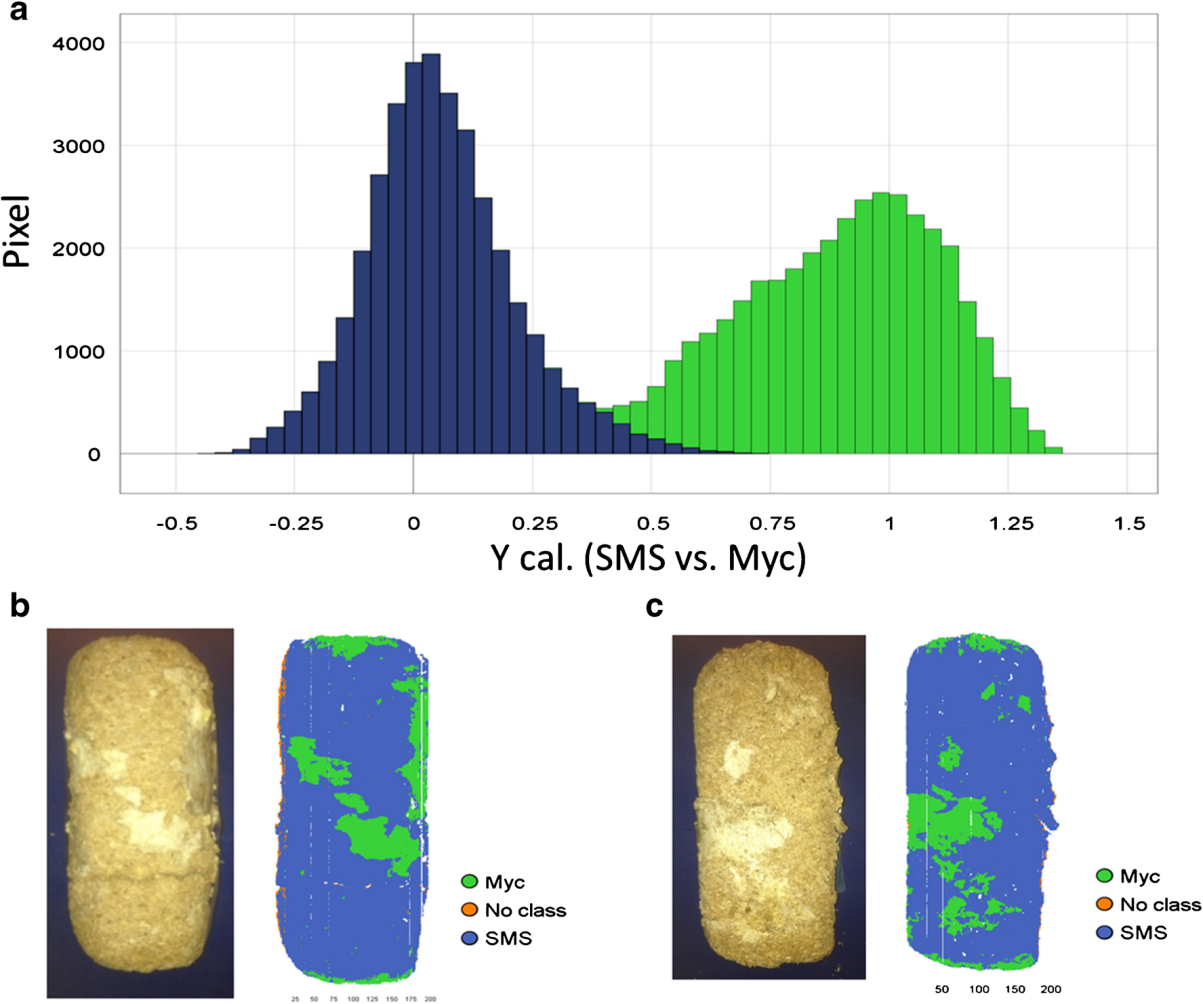
Partial least squares discriminant analysis (PLS-DA) modeling and prediction based on the dry material in setup I. **a** The histogram of the model prediction of the supervised pixels for spent mushroom substrate (*SMS*, *blue*) and mycelia (*Myc*, *green*) from the training set. Two classes were well separated, and the slight overlap ranged from 0.375 to 0.75. Misclassified pixels accounted for 0.6% of the whole data set. The second run of Exp. 1 (**b**) and the third run of Exp. 2 (**c**) were used as prediction sets. SMS and Myc were predicted correctly by the model

PLS-DA, as one of the approved methods for classification, was used to detect and confirm the results of PCA modeling based on images from setups I and II (Figs. 3 and 4). The training sets and prediction sets constructed for modeling are listed in Table 2. No cross-validation method was chosen because the data cube is very big and test prediction sets were used from the same experiment. The pixel clusters of SMS and Myc within the score plots (Figs. 3a and 4a) were selected as two classes and set to have equal size. Within the training sets, 72,808 and 276,655 pixels were chosen for model 1 and model 2, respectively. Three components were calculated for PLS-DA models, and they explained 85 and 77% of SS (the sum of squared differences from the mean) of the *y*-variables (SMS and Myc) for setup I and setup II, respectively. More than 99% of supervised pixels for SMS and Myc within the training sets were predicted correctly by two models (Table 2). Only 0.6% of pixels from model 1 and 0.3% from model 2 were misclassified. Better separation was found for the dry material (model 1, Fig. 5a) than the wet and plastic covered material (model 2), and two classes of model 1 were slightly overlapping within the prediction histogram of the supervised pixels. The region extended from 0.375–0.75 (Fig. 5a), while the overlap was found at 0.4–0.75 for model 2 (not shown in figures). When the models were used to predict the unknown samples from the same experiment, 99% of pixels from Exp. 1 and 2 and 98% of pixels from Exp. 3 and 4 were correctly predicted (Table 2, Fig. 5b, c) by models 1 and 2, respectively, which confirmed that the correct pixel clusters for SMS and Myc were chosen by PCA modeling. The loading peaks in Figs. 3c and 4c indicated that there was chemical variation between them.

**Table 2:** Summary of PLS-DA models

Content	Model 1: dry materials	Model 2: wet and plastic covered materials
Training set	Exp. 1 and 2 (3 runs)	Exp. 3 and 4 (4 runs)
Classes identification	Pixel clusters in the PCA score plot	Pixel clusters in the PCA score plot
Classes	2 (SMS and Myc with equal data size)	2 (SMS and Myc with equal data size)
Number of pixels	72,808	276,655
No. of wavelength	246 (949–2476 nm)	246 (949–2476 nm)
Pre-processing	SNV + center	SNV + center
Cross validation	Null	Null
No. of PCs	3	3
R^2^y_cum	0.851	0.772
Model sensitivity		
SMS	Pixel num.: 36,400; correct: 99.0%	Pixel num.: 135,462; correct: 99.8%
Myc	Pixel num.: 36,374; correct: 99.5%	Pixel num.: 137,754; correct: 99.5%
Misclassified	0.6%	0.3%
Prediction set	Predicted pixel (%)	
Test prediction	Exp. 1 (1 run: 99.1)	Exp. 3 (1 run: 98.0)
	Exp. 2 (1 run: 99.7)	Exp. 4 (1 run: 98.5)
The plastic cover effect	–	Exp. 5 (1 run: 94.8)
	–	Exp. 6 (1 run: 94.2)
Spectra representativeness	Exp. 7 (3 runs: 99.8)	Exp. 9 (3 runs: 90.1)
	Exp. 8 (1 run: 99.9)	Exp. 10 (1 run: 81.3)

*PCA* principal components analysis, *PCs* calculated principal component, *SMS* spent mushroom substrate, *Myc* mycelia, *SNV* standard normal variate transformation

### Chemical differentiation between SMS and Myc

**Fig. 6:**
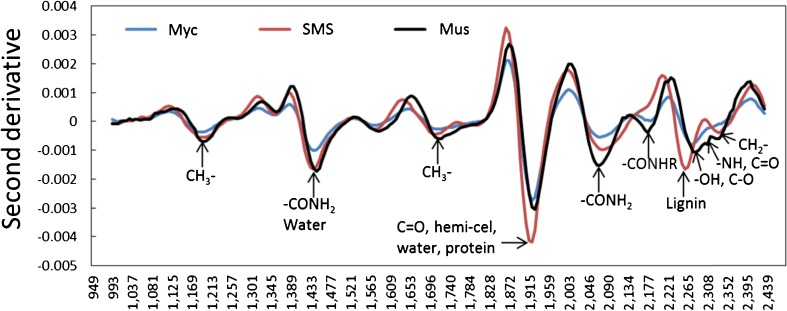
The second derivative of mean spectra of spent mushroom substrate (*SMS*) and mycelia (*Myc*) based on pixel cluster identification of the dry materials in Fig. 2. The mushroom (*Mus*) was adopted as a chemical reference. The chemical assignments of the wavelengths are from previous studies [8, 17]

Comparable peaks of the mean spectra for SMS and Myc clusters of the dry materials were identified by calculating their second derivative. The literature data was used from Osborne et al. [8] and Schwanninger et al. [17] for the chemical assignment of wavelengths of specific negative peaks of the second derivative in Fig. 6. To confirm the assigned peaks, mushroom was adopted as a reference because of widely available associated chemical information. SMS, Myc, and mushroom shared same absorbance peaks assigned to carbohydrates (1184–1200, 1358, and 1703–1721 nm) and protein (1439–1446, 1577–1590, and 2065–2090 nm) (Fig. 6). According to the peak intensity, mushroom had a higher protein content (2065, 2177, 2294, and 2308 nm), while SMS had higher hemicellulose/organic acid (1922 nm) and lignin contents (1790 and 2258 nm). Similar trends were found for Myc and mushroom, but less protein was found in Myc than mushroom. Less hemicellulose and lignin but higher protein content was found in Myc than in SMS, and this was consistent with the strong peaks (positive effects caused by Myc at 1922–2046 nm and negative effects caused by SMS at 2233–2265 nm) for the second loading of PCA modeling in Fig. 3c, which successfully separated SMS and Myc into different pixel clusters. These findings are considerably consistent with the results from previous scientific reports [11–13, 18]. Laminarin was found as the major component of fruit bodies and mycelia cell walls in *P. ostreatus*, and the main chemical compounds of fruit bodies and pure mycelia were carbohydrates (55–69 and 50% of total dry weight, respectively), protein (18.8–24.3 and 23.3%), ash (6.1–8.0 and 5.8%), and fatty acids (1.6–3.5 and 1.5%) [11, 12]. There were comparable glycogen [13] and chitin [18] contents in mushroom and mycelia, and glucoside was the major unit of cellulose, laminarin [19], and chitin [20]. Thus, it is not difficult to understand why SMS, Myc, and mushroom shared absorbance peaks associated with carbohydrates.

### Effects of moisture and the plastic covering

**Fig. 7:**
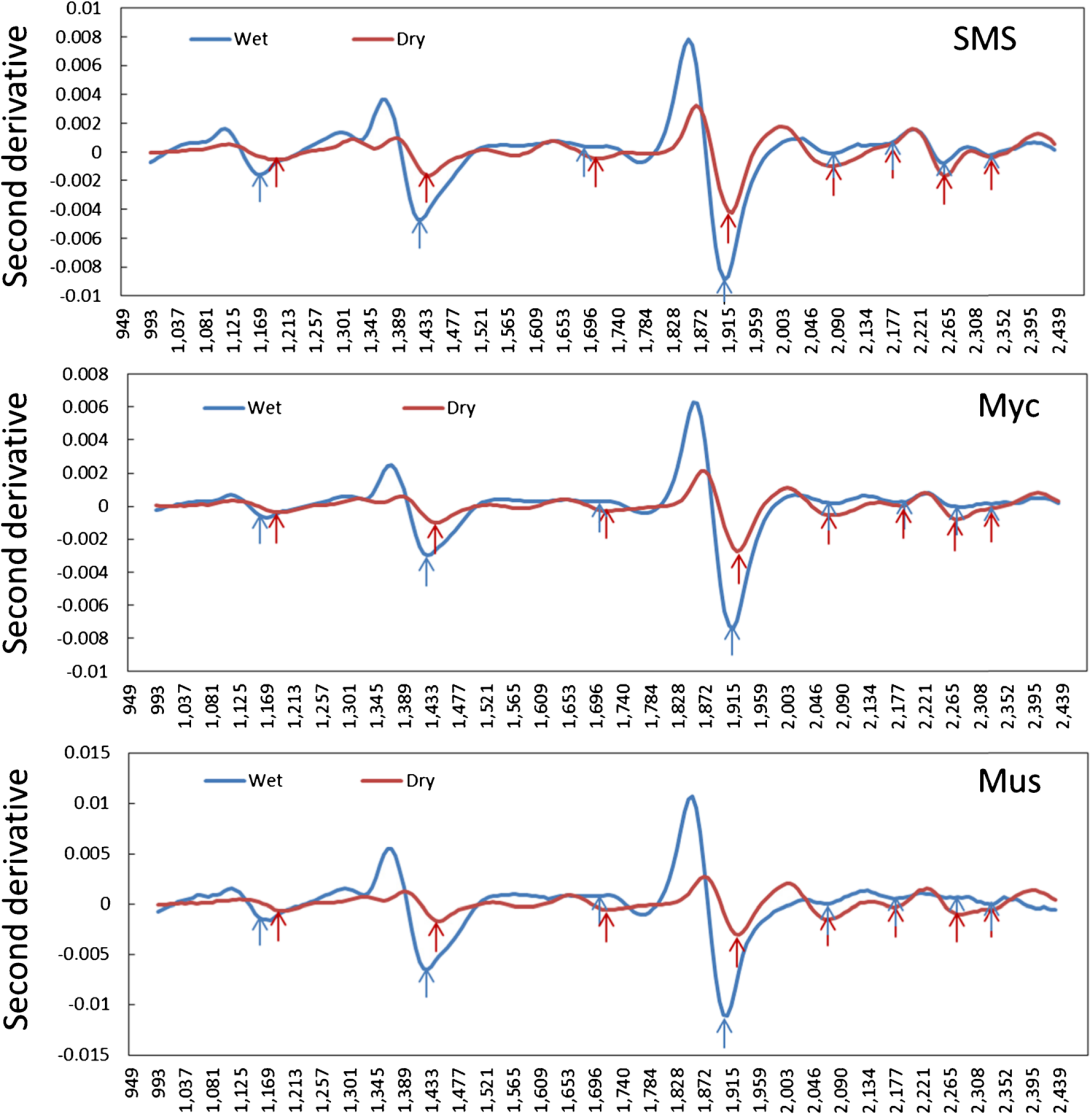
The second derivative of average spectra of spent mushroom substrate (*SMS*) and mycelia (*Myc*) based on pixel cluster identification of the dry (Exp. 1 and 2) and the wet (Exp. 5) materials. The mushroom (*Mus*, Exp.13) was adopted as a chemical reference. Peak shifts were found at a wavelength range of 1000–2000 nm

Mushroom substrates were packed within plastic bags during the mushroom cultivation process and the moisture content of spent substrates is about 65%. To characterize the chemical compound of the spent substrate with less sample preparation would be ideal for its utilization in decision-making. According to the results of PCA modeling, high moisture content caused the strongest effect on the SMS spectra. Thus, it is important to investigate how moisture affects the spectra. The effects of moisture were identified by comparing the negative peaks of the second derivative of average spectra for SMS and Myc under wet (Exp. 5) and dry (setup I) conditions, respectively, while the dry materials were treated as an ideal case for substrate chemical characterization. Using mushroom as a chemical reference, the spectra of the wet materials were illustrated by strong negative peaks at 1400 and 1920 nm (Fig. 7), which could be assigned as water molecular bonds shared with other bonds shown in Fig. 6. Furthermore, the comparison of the dry and the wet SMS and Myc (Fig. 7) indicated that apparent peak shifting at wavelengths of 1000–2000 nm caused by moisture was observed while there was little influence on the wavelengths above 2000 nm. This is consistent with the results of PCA modeling (Figs. 2 and 3) and explains why the strong loading value peaks of the wavelength in Figs. 2c and 3c were slightly different (1959 and 2246 nm for the dry material and 1922 and 2240 nm for the wet and plastic covered materials). However, with regard to the dried materials (red arrow in Fig. 7), the negative peaks assigned as carbohydrates, protein, and lignin can still be clearly identified (blue arrow, Figs. 6 and 7). For the calibration of chemical content in wet materials, good NIR calibration models have been established to predict water, fat, and protein contents in lamb meat under 70–76% moisture content conditions by Kumruzzaman et al. [21] via an NHI system in the NIR spectral range of 900–1700 nm. Thus, an NHI system with a larger spectral range of 900–2500 nm, which could explain more chemical information with no moisture effect at 2000–2500 nm than the one with wavelengths 900–1700 nm, has great potential for making calibration models to predict the water, carbohydrate, lignin, and protein contents of the wet SMS.

**Fig. 8:**
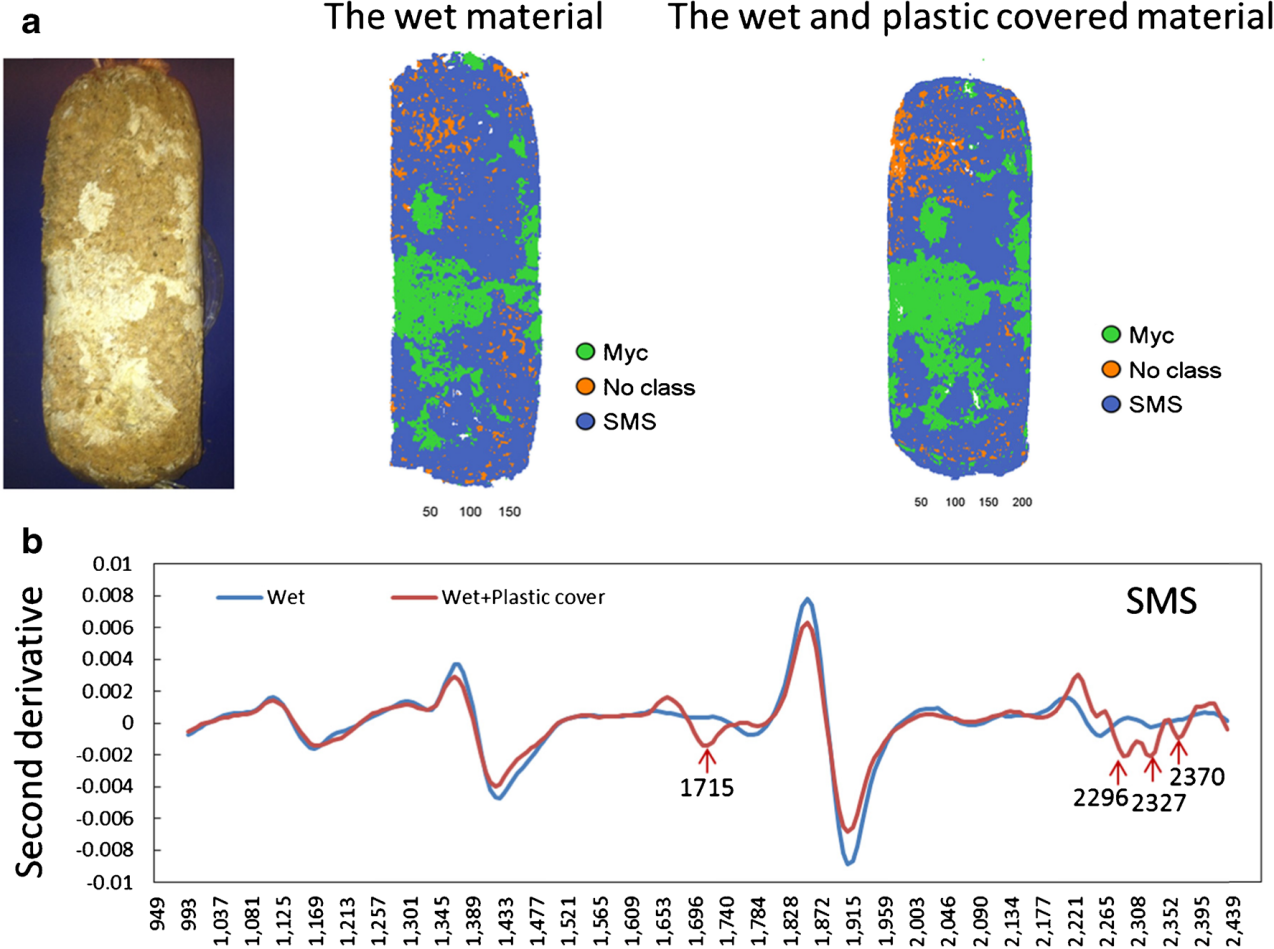
Effects caused by the plastic cover on wet material. **a** The digital image (*left*) and predicted mosaics of the same material under the wet (*middle*) and the wet and plastic covering (*right*) conditions by the partial least squares discriminant analysis model based on setup II. Most of the spent mushroom substrate (*SMS*) and mycelia (*Myc*) were predicted correctly. **b** The second derivative of the mean spectra of SMS under different conditions. Even though four unexpected peaks were created by the plastic chemical contamination, the important peaks for water, carbohydrates, protein, and lignin can still be identified for the plastic cover SMS

To investigate the effect of the plastic cover on the wet substrate, the second PLS-DA model in Table 2 was applied to predict the SMS mosaic for the same object’s surface without and with the plastic cover treatments (Exp. 5 and 6). About 94% of SMS and Myc was predicted successfully, and there were comparable predicted results for both experiments (Table 2, Fig. 8a). The result indicated that the plastic cover caused less influence on spectra than the difference in SMS and Myc, which was consistent with the loading peaks (1715, 2298, 2335, and 2373 nm) of overview PCA modeling (Fig. 2d). The plastic cover effect was examined by comparing the second derivative of the mean spectra of SMS under the wet and plastic covered conditions (Fig. 8b). Although four unexpected peaks were caused by plastic chemical contamination, the important peaks for water, carbohydrates, protein, and lignin can still be identified for SMS covered by plastic. Similar results were gained for Myc and mushroom as well (not shown in figures). As indicated by the PCA modeling (Fig. 2), moisture caused stronger effects on the spectral data set than the chemical difference between SMS and Myc and the influence caused by the plastic cover when the moisture content was approximately 65%. All these results suggested great potential for water and other organic compound calibration modeling for SMS by using NHI without removing the plastic cover of the wet SMS cylinder. However, the Myc should be removed in advance via MIA.

### The representativeness of SMS spectra

Research on embryo visibility in the maize kernels by NHI [9] indicated that the penetration depth of the NIR radiation is about 1–2 mm under the object’s surface. This implies a possibility that the representativeness of the SMS information gained is not good enough due to the heterogeneous surface, which might influence the NIR penetration when imaging on the side surface of the cylinder. In the current study, the cross section of the cylinder and the bulk-mixture treatments are considered as good representative samples of the SMS cylinder, and their NIR spectra were compared with those from the side surfaces. To compare the spectra of different profiles, PLS-DA models constructed on the images taken on the side surfaces of the cylinder under the dry and the wet and plastic-covered conditions (Table 2) were applied to predict the mosaics from the cross section (Exp. 7 and 9) and the bulk mixture (Exp. 8 and 10), respectively. The results showed that more than 99% of pixels of the dry materials were predicted, and 81–90% of pixels of wet material were predicted correctly (Table 2). The prediction result for the wet materials was not as good as the result for the dry material because the plastic cover effects were included within the training set of the PLS-DA modeling. These results suggest that the penetration depth of light radiation is enough, and making images on the side surface could represent the information for the whole SMS cylinder, as long as the substrate is reasonably homogenized. Therefore, it is possible to get a representative spectrum for SMS when making hyperspectral images on the side surface of the SMS cylinder, even when it covered with plastic.

It is important to consider that the chemical composition of the substrate is changing while mushrooms are growing, and suitable moisture content of the substrate is one of the most essential factors for mushroom growing. The carbohydrate and lignin levels of SMS will affect feedstock quality for biofuel production. Thus, collecting SMS on time is very important for avoiding the excessive degradation of substrates by mycelium respiration, which could maintain the feedstock quality of SMS for bioenergy. Compared to wild mushrooms, mushroom cultivated on the substrate is a controllable system. Building up a good system for creating an optimal environment for mushroom growing and preserving the energy of the substrate as much as possible would be ideal for the mushroom industry. It is important and worth trying to develop NHI on-line technology, not only to analyze moisture content but also for chemical composition, controlling substrate quality for mushroom cultivation and biofuel production. To achieve this in the future, a number of responses, such as protein, carbohydrate, and lignin, should be analyzed via wet chemical analysis and recorded for quantitative calibration modeling.

## Conclusion

This study aimed to investigate the possibilities of using NHI and MIA as an on-line and nondestructive method for chemically analyzing SMS, which is very heterogeneous in structure with high moisture content and plastic packing. Overview PCA modeling indicated high moisture content caused the most significant effects on spectra followed by the uneven distribution of Myc and the plastic cover. Both PCA and PLS-DA modeling were applied to classify SMS and Myc clusters and the classification showed similar trends, whether the materials were wet or dry. The loading peaks from PCA modeling and the second derivative-corrected spectra for SMS and Myc clusters also showed similarities in most peaks with rather clear differences in chemical composition. MIA can be used to spatially remove the effect caused by chemical differences between SMS and Myc by easily excluding the Myc pixel cluster within the PCA score plot. The best results for classification and the chemical identification of SMS were obtained for dried cylinders, but it was shown that almost equally good results could be obtained for wet material and for wet material covered by plastic. Therefore, the combination of NHI and MIA has great potential to make calibration models, using side surface images after the removal of Myc to predict the contents of water, carbohydrates, lignin, and protein in wet and plastic-covered SMS cylinders.
